# Implementing Industry-Inspired Quality Practices in Healthcare: A Study From the Pathology Department of the University Hospital Center of Tangier

**DOI:** 10.7759/cureus.71377

**Published:** 2024-10-13

**Authors:** Jamal Essraidi, Mariame Chraibi

**Affiliations:** 1 Faculty of Medicine and Pharmacy of Tangier, Abdelmalek Essaadi University, Tangier, MAR; 2 Department of Pathology, University Hospital Mohammed VI of Tangier, Tangier, MAR

**Keywords:** join commission international, pathology, pdca cycle, quality management system, risk analysis matrix

## Abstract

In Morocco’s public health sector, scientific research has produced only theoretical models of quality, with empirical studies sometimes producing practical recommendations. In contrast, benchmarks from the industrial sector demonstrate a much smoother and more effective implementation of a quality culture. This article considers practices inspired by the quality culture in the industrial sector, applying them in the Department of Pathological Anatomy and Cytology (ACP) of the University Hospital Center of Tangier (CHUT). The ACP department has demonstrated a strong commitment to fostering a dynamic quality culture in its professional activities, focusing on the advancement and regulation of pathology techniques to enhance procedures and deliver accurate diagnoses to patients. This approach could facilitate the implementation of quality systems management that would earn certification and/or accreditation. Our methodology applied the “Plan-Do-Check-Act” and “Standardize-Do-Check-Act” models to analyze the initial quality and compare this with the Join Commission International standards chosen by CHUT. We then planned the implementation of corrective actions for each deviation and verified the efficiency of the action plan, and finally maintained a dynamic cycle. There was an implementation score of 60% in two years, mainly starting with the development and implementation of risk analysis for all activities of laboratory development and the implementation of specific Key Performance Indicators KPI and specific methods to manage all human resources items.

## Introduction

Since January 2021, the Pathological Anatomy and Cytology (ACP) Department of the University Hospital Center of Tangier (CHUT) has been engaged in a project to implement quality practices inspired by those used in the industrial sector. The primary aim of this project was to integrate quality practices into the ACP department, ensuring the traceability of various techniques, improving results, and achieving accreditation in the coming years. The goal is to meet the international standards such as the Join Commission International (JCI), which have been adopted by CHUT. This initiative is the first of its kind in Morocco. 

To achieve this objective, our work focused on identifying where the ACP practices diverged from JCI standards, establishing corrective action plans, and ensuring the implementation of those plans in the department. In this article, we present the three most important axes that have recovered more than 60% of our action plan and meet 80% of JCI’s requirements. The first axe is intended to manage the competencies of laboratory staff through the implementation of a skills-management matrix [1]. All laboratory staff currently have the competencies needed to develop a quality culture in the department. The qualification of human competencies is the most important step [2], preventing resistance to change [3] during the implementation of the quality management system (QMS). This QMS represents the latest evolution of the quality concept [ 4]. The second axe included the key performance indicators (KPIs) that provide the road map for indicators of ACP service, and the third axe was for the identification of all risks in the department, the analysis of that risk, and setting up the related action plan for all deviations. These three areas are the focus because they accord with six aspects of healthcare: equity, safety, efficacy, patient-centeredness, timeliness, and efficiency [5]. In the industrial sector, the pursuit of quality is the priority in each of these six domains [6,7]. Thus, in this study, the quality enhancements in healthcare were modeled on those of these businesses. Despite the clear distinctions between these industries and healthcare, there are lessons for the latter to learn from the former [8]. Thus, our work took inspiration from the “quality culture” in the industrial sector in developing tools for use by the ACP department of CHUT. These tools included a method of managing human resources, a dashboard KPI that provides a road map for the service, and a risk-analysis matrix. These three tools recover more than 60% of our action plan.

## Materials and methods

**Figure 1:**
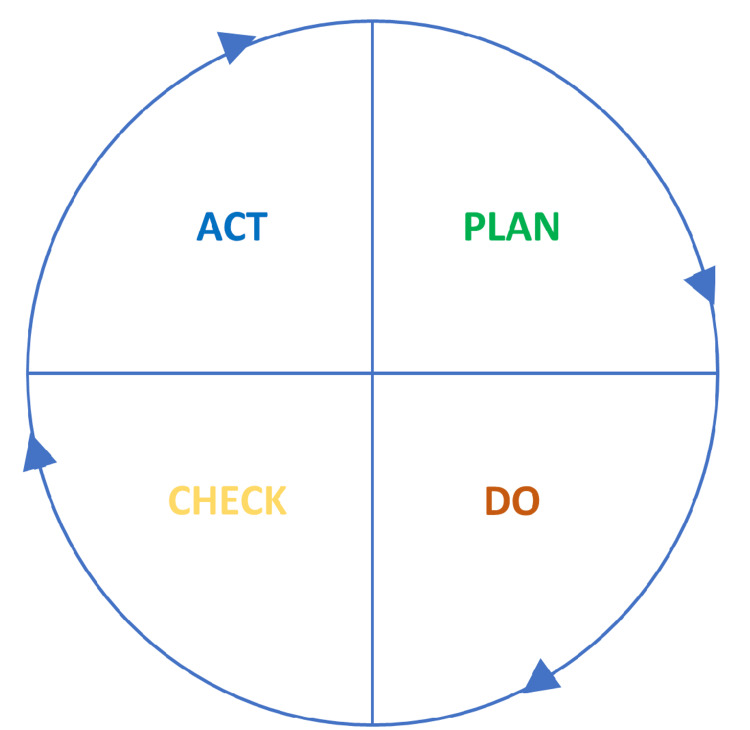
The PDCA cycle Diagrammatic representation of Plan–Do–Check–Act (PDCA) cycle (Image credit - Jamal Essraidi, Mariame Chraibi).

Our project used the JCI 3rd edition as a reference to assess the existing quality system in the ACP department [9]. We used the Plan-Do-Check-Act (PDCA) cycle [10] to highlight any major gaps, analyse the root cause of the deviations, correct them, and make continuing improvements. This systematic approach was developed based on experiences in domains such as industry [11-13], business processes [14], and medical record quality management [15]. The PDCA, also named the “Deming Cycle,” was developed by William Edward Deming and comprises 14 principles that became the basis of the philosophy of quality in organizations and is employed to facilitate continuous improvement [12]. The PDCA cycle is extremely versatile and can be used successfully with any type of business [16]. It provides a visual representation of the process of continuous improvement, comprising four successive and sequential stages: planning, doing, checking, and acting (Figure 1).

**Figure 2:**
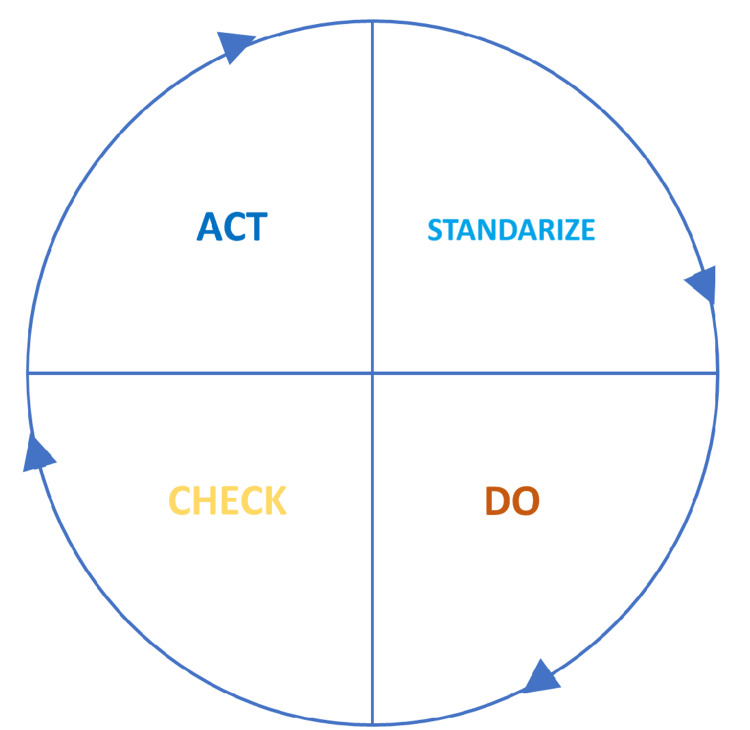
The SDCA cycle Diagrammatic representation of Standarize-Do–Check–Act (SDCA) cycle (Image credit - Jamal Essraidi, Mariame Chraibi).

The PDCA method can be applied to almost any situation in a company, independent of the domain or activity profile of that organization. However, no quality system had been implemented in the ACP department of Tangier prior to this initiative, with the exception of some written standards. To apply the PDCA cycle, it was thus essential to create the standards and guarantee their related stabilities, as recommended by Knop and Mielczarek in their paper [16]. The process stabilization is the Standardize-Do-Check-Act (SDCA) cycle [16] (Figure 2). The SDCA methodology maintains continuous improvement through the execution of four stages: Standardize, where the target standards are defined; Do, where the standards are implemented; Check, where the actual work is compared to the standards; and finally, Act, where any variations from the standards are reviewed and addressed [16].

After the appropriate method had been chosen, the second step was to prepare the laboratory staff, ensuring they had the competencies and skills needed for implementation and the motivation to engage with the project. This involved various practical training sessions on applying quality tools such as Preto and Ishikawa’s diagram, the PDCA and SDCA models, and the JCI norms.

In the third step, we conducted a collective audit of the initial situation, drawing comparisons with the JCI norms and focusing primarily on laboratory standards. The relevant JCI norms are given in Table 1.

**Table 1:** Representation of requirement categories as represented in the JCI norms JCI: Join Commission International.

Section	Requirments categories
Section I	Accreditation Participation Requirements.
Section II	Patient-Centered Standards International Patient Safety Goals.
Section III	Health Care Organization Management Standards Governance, Leadership, and Direction - Management of Information - Staff Qualifications and Education - Facility Management and Safety - Quality Control Processes.

For each requirement, we checked if the deviation occurred because there was no related standard, the existing standard was inadequate, or the relevant standard was not respected. We categorized the deviations (and related corrections) as follows (Table 2).

**Table 2:** Representation of deviation categories based on priorities JCI: Join Commission International; PDCA: Plan-Do-Check-Act; SDCA: Standardize-Do-Check-Act; ACP: Pathological Anatomy and Cytology.

Deviation Category	Priority
Standard applied but needs to be aligned with the JCI. (PDCA cycle to be applied.)	Priority 1
No standards exist, (SDCA cycle to be applied.)	Priority 2
No deviation occurred, but the situation needs improvement. (PDCA cycle to be applied.)	Priority 3
Standards not relevant to the ACP department.	Priority 4

The work was conducted with the support of the ACP department team. Using the PDCA or SDCA models, we analysed the gaps and implemented corrective actions to meet the related requirements. To ensure the deployment of the action plan, we launched pilots and set deadlines for each action. The effectiveness of the corrective actions was then measured after implementation. To illustrate our article, we presented the action plan form in Table 3 below.

**Table 3:** Global action plan for deviations from JCI standards, as identified from the first check JCI: Join Commission International.

Items	Standard Number	Priority	Gap	Action	Pilot	Deadline
Section I: Accreditation Participation Requirements (APR)	Requirement: APR.1					
	…					
	Requirement: APR.12					
Section II: Patient- Centered Standards "International Patient Safety Goals (IPSG)"	Standard IPSG.1					
	…					
	Standard IPSG.5					

## Results

**Figure 3:**
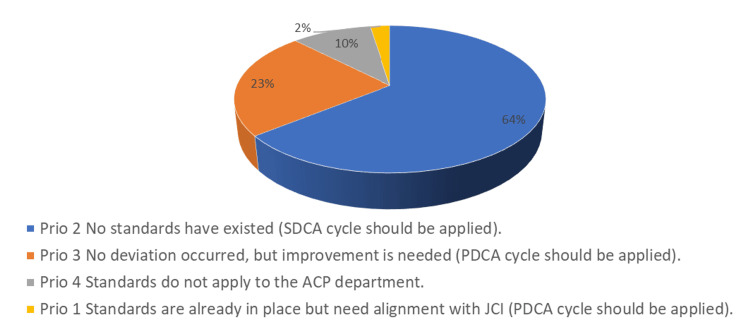
Result of initial check according to JCI standards JCI: Join Commission International; Prio: Priority; SDCA: Standardize-Do-Check-Act cycle; Plan-Do-Check-Act cycle (PDCA); ACP: Anatomy and Pathological Cytology.

The initial results indicated that more than 64% of the standards were classified as “Prio 2,” which means the ACP department had no standards meeting the JCI requirements (Figure 3).

To apply the SDCA, we began by developing the standards needed to meet the JCI requirements, taking lessons from the industrial sector, and adapting those tools to suit the ACP department. Many of the deviations concerned the competencies and skills of the people in the department, with changes needed to their responsibilities, leadership functions, qualifications, knowledge and experiences, and education and training. More than 24% of the JCI requirements for laboratories concern the competencies and skills of the people [1].

**Figure 4:**
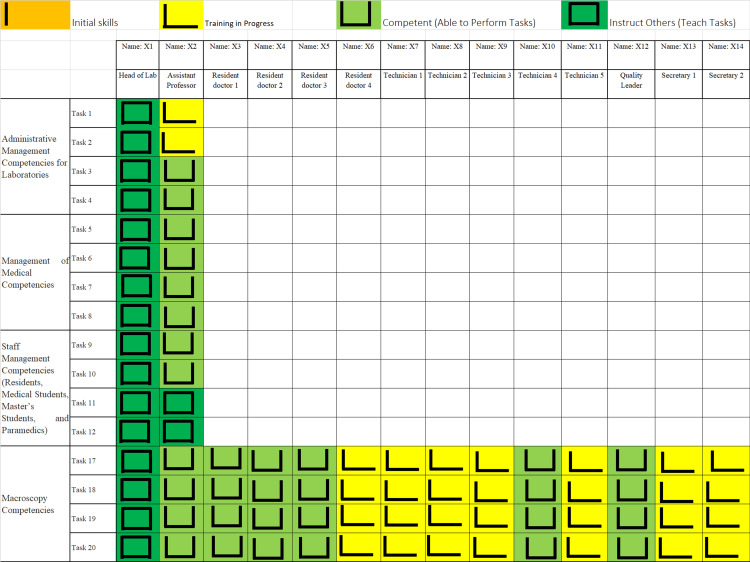
ILUO matrix of the management of competencies and skills in the ACP department I: A beginner without skills; L: In training; U: Trained; O: An expert; ACP: Pathological Anatomy and Cytology.

The first tool developed was a skills-management matrix named the “ILUO” method [1], which categorized the competencies of department staff into four levels according to their qualifications: “I,” a beginner without skills; “L,” in training; “U,” trained; and “O,” an expert able to teach others. Illustrating the ILUO method, Figure 4 below presents the formal standard adopted in the department as an example given here without real data in order to preserve confidentiality.

The ILUO matrix enables a visual assessment of the competencies and skills to be developed, binding the quality-system requirements with the human resource items. This enables top management to identify non-conformities and deficiencies in competencies and establish training plans for department staff to ensure continuous improvement [1]. 

**Figure 5:**
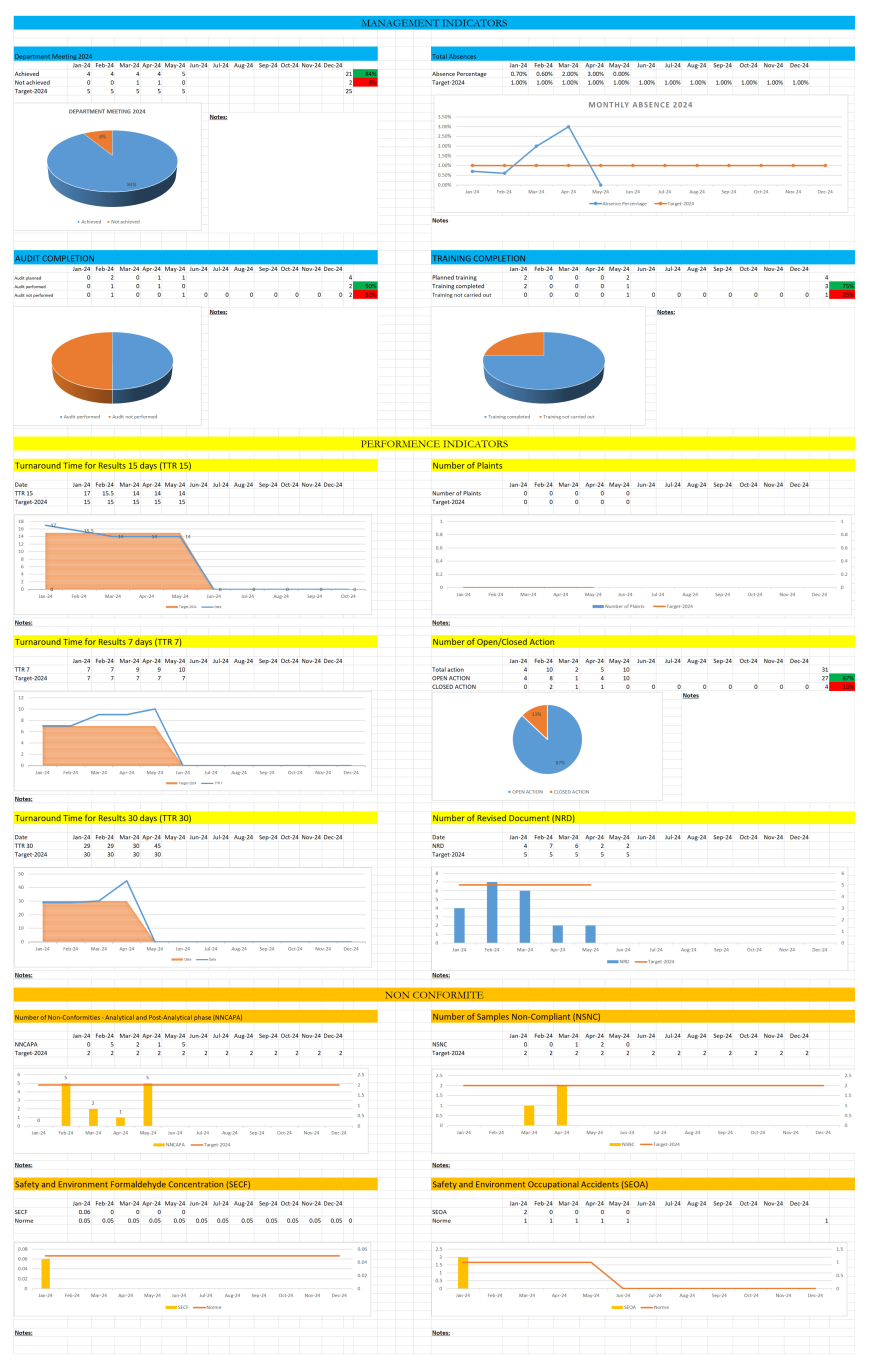
Dashboard (KPI) developed for the ACP department in CHUT CHUT: University Hospital Centre of Tangier; ACP: Anatomy and Pathological Cytology; KPI: Key Performance Indicators; NRD: Number of Revised Document; TTR 15: Turnaround Time for Results 15 days; TTR 7: Turnaround Time for Results 7 days; TTR 30: Turnaround Time for Results 30 days; NNCAPA: Number of Non-Conformities - Analytical and Post-Analytical phase; NSNC: Number of Samples Non-Compliant; SECF: Safety and Environment Formaldehyde Concentration; SEOA: Safety and Environment Occupational Accidents.

The second tool developed for this study was a key performance indicators KPI tailored to the needs of the ACP department (Figure 5).

To preserve the confidentiality of the department, the simulation of the KPI in this article does not include real data. This dashboard incorporates quality management indicators, follow-up from department meetings, audit planning, staff training, and absence rates. It also includes performance indicators such as the time of obtaining results and the follow-up of non-conformities. The KPI was updated monthly by the staff using real data, with the data then analyzed to determine the need for corrective action around any deviations or nonconformities.

The third tool developed in our work is the risk analysis matrix designed for the ACP department. It is presented in a large table, with a small section provided here to demonstrate the overall structure of the risk analysis, Table 4.

**Table 4:** Example of risk analysis matrix for the ACP department of CHUT CHUT: University Hospital Centre of Tangier; ACP: Anatomy and Pathological Cytology; RPN: Risk Priority Number.

Process step	Task	Classification	Potential Failure Mode	Potential Effect(s) of Failure	Severity	Potential Cause(s)of Failure	Current Process (Moyen de maitrise)				RPN	Recommended Action	Responsibility & Target Comletion Date	Action Taken Completion Date	Severity	Occurance	Detection	NEW RPN*
								Occurance	Current Process	Detection								
							Current Process											
																			Reception of samples	Preparation of vials for the ACP laboratory	P	Non-conforming fixative	Patient dissatisfied, no results	7	Operating error	Instructions to prescribing physicians (use of 10% buffered formalin)	0.5	check the faisability of detecting Formol	0.5	1.75							
																					P	Fixing delay	Patient dissatisfied, no results	7	Operating error	Circulation of instructions to prescribing physicians	0.5	Requiring the date and time of sampling on the clinical report form	0.5	1.75							
																					P	Fixation of samples for extemporaneous examination, immunofluorescence or PCR techniques	Patient dissatisfied, no results	7	Operating error	instructions for prescribing physicians (do not fix a sample intended for extemporaneous examination, immunofluorescence or PCR techniques)	0.5	Visual control	0.5	1.75							
Training problem																																					
Transfer and transport of vials	NA	Contaminated bottles or accompanying documents	Inflammation caused by fixative.	1	Bottle opening during transport	Suitable, tightly sealed bottles	1	Visual control	1	1									
			Irritation of respiratory tract																
			Ocular irritation																
	P	Non conforming bottle (Vials) / not tightly sealed	Loss of the sample.	7	Operating error	instructions for prescribing physicians (to us suitable and tightly sealed vials / to use vials large enough to prevent distortion of bulky surgical parts) Use of transparent plastic bags	1	Visual control	0.5	3.5									

Table 4 was developed using a form of the “failure modes, effects, and analysis” (FMEA) process, adapted for the ACP department. All risks were identified at every stage of the department's process, as well as during the preanalytical, analytical, and post-analytical processes [17]. Table 4 identifies failure modes, their potential effects, and classifications based on patient-centered aspects (P), along with the severity of these effects, their causes, frequency, detectability, and the corresponding Risk Priority Number (RPN). The table’s empty columns are typically filled in when RPN analysis is required, followed by the implementation of corrective actions. These actions must include detailed descriptions, target responsibilities, completion dates, and a reassessment of severity, occurrence, detection, and RPN. These risks were analyzed by the department team and assessed according to an internal grid, as presented in Table 5.

**Table 5:** Detection, severity, and occurrence assessment grid

	Factor	Interpretation
Severity	1	Minor: No injury, no treatment
	4	Major: Needs care/difficulty of interpretation
	7	Serious: Delay in care
	25	Critical: Inability to work
	40	Catastrophic: Fatal
Occurrence	0.5	Rarely
	1	Sometimes
	2	Frequently
	6	Continuously
Detection	0.5	Easy to detect
	1	Difficult to detect
	3	Undetectable

The risk priority number (RPN) is fixed at 30 to prioritize all risks that need to be corrected. The team then analyzed the risks, established appropriate corrective and preventive actions, and ensured a control plan for the department processes.

## Discussion

The originality of our work is the integration of quality practices into the daily routines of the pathology department staff at CHUT, achieved without the need for specific investments or additional time, drawing inspiration from the quality culture of the industrial sector. By utilizing KPIs, a risk-analysis matrix, and the ILUO skills matrix, we created an environment conducive to the implementation of quality systems, regardless of the standards being applied, including the International Organization for Standardization (ISO) 9001, ISO 15189, ISO 17025, and JCI. All these references aimed to ensure the quality of services and techniques, each with its own specific features [18]. They required the use of a KPI dashboard to monitor quality systems, a risk-analysis matrix to identify, analyze, and address risks, and the ILUO method to manage competencies effectively.

This work refers to the JCI norms because these are CHUT’s chosen quality targets and are applied in all of its healthcare organizations, including hospitals, clinics, and laboratories. They cover all aspects of health care and are not focused solely on laboratory activities as ISO 15189 and ISO 17025 [19].

The three practical tools developed for this study were incorporated into the daily tasks of the team. The tools met the requirements of the KPI by including the results of identified indicators, enabling the analysis and setting up of corrective actions for all deviations, and monitoring continuous improvement. We updated the risk-analysis matrix when new risks were identified and/or when the RPN needed to be analyzed. A preventive action plan was then put in place to ensure the avoidance of pitfalls. These practices became easier for the team as we worked on the management of staff competencies and skills to optimize the resources and develop training plans.

Our strategy prioritized the implementation of practical quality tools over an initial focus on documents and procedures. Excessive bureaucracy can hinder the development of new practices and increase costs, ultimately compromising the quality of patient care [18]. Some pathologists view certain requirements, like the ISO 15189 standard, as challenging to integrate into the daily operations of the laboratory and the diagnostic activities fundamental to their profession [20].

Despite our tangible results, we faced several limitations, including the absence of a dedicated budget for the project, which is particularly challenging for the next steps in implementing the quality management system where certain actions require investment. Additionally, we encountered a lack of full engagement from all services surrounding the pathology department, which is crucial for establishing a unified process across the entire hospital.

The accreditation of pathology services lacks global standardization, leading to significant variations between countries [18]. In Morocco, there are no specific laws or regulations governing the field of pathology. As a result, diverse auditing approaches and varying interpretations of adopted norms are prevalent. This article, therefore, emphasizes the development of a quality culture in daily practices to ensure service quality, minimize false-negative and false-positive results, and optimize patient treatment time.

## Conclusions

The development and implementation of the quality tools discussed in this article-namely, the KPI, the risk-analysis matrix, and the ILUO skills matrix method have provided the CHUT ACP department with a quality culture that could also be adopted by other CHUT services and public health facilities. However, this innovative approach presents several new challenges, such as the need to support various techniques involved in the quality approach, to manage processes and related risks, to monitor the quality indicators, and finally, to ensure continuous improvement despite the personnel and material constraints.
